# Behavioral and Neuropsychological Evaluation of Executive Functions in Children with Autism Spectrum Disorder in the Gulf Region

**DOI:** 10.3390/brainsci10020120

**Published:** 2020-02-22

**Authors:** Rehab H. Alsaedi, Suzanne Carrington, James J. Watters

**Affiliations:** 1 Faculty of Education, Queensland University of Technology, Victoria Park Road, Kelvin Grove, Brisbane, QLD 4059, Australia; sx.carrington@qut.edu.au (S.C.); j.watters@qut.edu.au (J.J.W.); 2 The Department of Special Education, Taibah University, Janadah Bin Umayyah Road, Madinah 41477, Saudi Arabia

**Keywords:** Executive functions, Autism, Gulf, BRIEF-2, CANTAB

## Abstract

This study examined the executive functioning abilities and development profiles of children with autism spectrum disorder (ASD). The participants were 119 children with ASD and 30 typically developing children (age range: 6–12 years) who were recruited from three Gulf states. The findings revealed executive functioning deficits in the ASD population when compared to the normative data or to those children without ASD. However, not all the forms of executive functioning were found to be impaired. Age-related differences in the patterns of performance on the utilized measures of executive functioning were also identified. The overall findings provide valuable information regarding the different components of the executive functions, which may prove useful in relation to the development of assessment protocols for ASD.

## 1. Introduction

The term “executive dysfunctions” refers to impairments in a range of loosely related cognitive processes that play a primary role in coordinating higher-order cognitive abilities and emotions, as well as in regulating behavioral responses to non-routine environmental demands [1]. Executive dysfunctions (EDFs) are among the most prevalent neurodevelopmental features associated with autism spectrum disorder (ASD) [2]. Indeed, it has been estimated that 41% to 78% of individuals with ASD exhibit EDFs [2]. Such deficits could hinder several areas of development, including children’s neurocognitive, behavioral and psychosocial development [3,4,5]. Due to the core or likely causal, role played by the executive functions in the presentation of the behavioral and social features of ASD, it has been suggested that executive dysfunction lies at the heart of the disorder [6,7]. EDFs are most commonly associated with abnormalities affecting the frontal lobe, particularly the prefrontal cortex (PFC), in both typically developing individuals and those with ASD [8,9]. However, it is now generally understood that the executive functions are not solely linked to the frontal brain structures but are instead associated with a widely distributed neural network and based on the integration of cortical and subcortical systems throughout the brain [10].

The literature reporting executive dysfunction to be a causal factor in ASD remains controversial. An accumulation of evidence has indicated that deficits in the executive functions should be considered central deficits in those with ASD [11,12], since such deficits are exhibited by individuals with ASD regardless of their age and functional level [13,14,15,16]. Additionally, executive functioning difficulties have also been identified in the parents and non-autistic siblings of children with ASD [17,18]. Yet, other studies have suggested there to be no primary executive functioning deficit in those with ASD, since such deficits are evident in older children with ASD but not in younger children [19]. Further, executive functioning deficits have also been reported in relation to other neurodevelopmental and neurological disorders, including schizophrenia [20], intellectual disability [21], Tourette’s syndrome [22], dyslexia [23] and attention deficit hyperactivity disorder (ADHD) [24].

Although individuals with ASD show a marked impairment in their executive functioning ability, neither all the executive functioning components nor all individuals with ASD are equally affected. It is difficult to determine exactly which EDFs are typical of ASD and which are not [9,25]. Numerous studies have indicated that flexibility and planning/organization represent the most commonly impaired executive functioning characteristics seen in individuals with ASD [9,26]. Some studies have suggested that inhibition control might be impaired in those with ASD [27], while others have proposed that the working memory [28], generativity [29] and self-monitoring [26] of children with ASD are impaired. Clearly, the evidence to date is inconclusive. The heterogeneity of the methods used in prior studies may have precluded the clear identification of the executive functioning deficits seen in those with ASD and it may also have contributed to the inconsistent patterns visible in the results thus far [9,30].

Opinions regarding the developmental nature of executive functioning in individuals with ASD are varied. Some studies have suggested the existence of age-related improvements in the executive functioning skills of individuals with ASD [31,32], while other studies have indicated that executive functioning deficits tend to remain stable in both children and adolescents with ASD [33,34,35,36,37]. However, the observed developmental improvements in executive functioning never reach normative adult levels [32]. Some studies have highlighted the importance of individually investigating the development of the different domains of executive functioning rather than considering the construct as a whole [38]. The executive functions may become increasingly differentiated with age, since they follow a multistage process involving different developmental trajectories [37,38,39] and it appears that the developmental patterns associated with executive functioning run parallel to growth spurts observed within the anterior brain regions [40].

Although executive dysfunction is a prominent theme in the ASD literature, there remains a paucity of studies examining executive functioning skills in Middle Eastern individuals, particularly those from Gulf Cooperation Council (GCC) countries (Bahrain, Kuwait, Qatar, Saudi Arabia, the Sultanate of Oman and the United Arab Emirates [UAE]), which did not recognize ASD until the late 1990s [41]. Thus, a significant need exists for more research regarding ASD and its manifest phenotypes, especially in terms of EDFs, which have vital implications for the taxonomy, assessment and diagnosis of the disorder, as well as for the design of treatment interventions.

The aims of the present study were threefold. First, to determine the prevalence of executive dysfunction in children with ASD, as identified using the Behavior Rating Inventory of Executive Function, Second Edition (BRIEF-2) [42] and to examine the executive functioning profiles of children with ASD relative to their typically developing (TD) peers. According to the BRIEF-2 manual, a score of 60 is regarded as the threshold for an abnormally elevated score. Based on the prior literature, it was hypothesized that the children with ASD would exceed the cut-off criteria for all the executive functioning domains of the BRIEF-2, with the most prominent impairment being seen in relation to the Shift domain. Additionally, it was hypothesized that the children with ASD would score significantly lower in relation to all the BRIEF-2 domains than their TD peers. Second, to examine the extent of the differences in executive functioning, as identified based on the Cambridge Neuropsychological Test Automated Battery (CANTAB) tasks [43], between children with ASD and typically developing children. As discussed earlier in this article, the particular executive functioning domains that are impaired (or preserved) in individuals with ASD remain unclear. Therefore, it was hypothesized that the children with ASD would exhibit executive dysfunction in relation to some (albeit not all) aspects of executive functioning. The third aim of the sub-study was to investigate the age-related differences (if any) in the executive functioning performance of the children with ASD. Due to the lack of consistent prior evidence, no hypotheses were formulated concerning the influence of chronological age on executive functioning in those with ASD.

## 2. Materials and Methods

### 2.1. Participants

Participants were recruited from three Gulf states, namely Bahrain, Saudi Arabia and the United Arab Emirates (UAE). Purposive sampling on a voluntary basis was employed. The initial study sample comprised 180 children with ASD (age range: 6–12 years) and 55 children with typical development. Of the 180 initial ASD cases, 13 (7.22%) participants were excluded from the study because their IQ scores were not available. Based on the scanning criteria, another 11 (6.11%) participants with ASD were excluded at the scanning stage. More specifically, despite having previously received a clinical diagnosis of ASD, these 11 participants failed to reach the cut-off point for ASD on the two screening measures used to confirm the clinical diagnosis in the present study. A further 12 (6.67%) participants with ASD were excluded because their parents did not attend the assessment stage. Another 24 (13.33%) participants with ASD were excluded because they were unable to complete the CANTAB tasks. Finally, one (0.56%) participant with ASD was excluded due to the effect of the amphetamine-based medication he had recently used. These exclusions resulted in a total of 119 (66.11%) participants with ASD being included in the final analysis. In terms of the children with typical development, the initial sample comprised 55 participants. Six (10.91%) participants with typical development who were thought to have learning difficulties were excluded from the study, while nine (16.36%) participants with typical development withdrew their consent to participate. Another ten (18.18%) participants had to be excluded because their assessments were incomplete, which left a total of 30 (54.55%) participants with typical development to take part in the analysis. Hence, the final study sample comprised 149 children who had previously been clinically diagnosed with ASD and 30 typically developing children. 

The participants with ASD were recruited from three different types of educational establishments, including a fully inclusive school setting (*n* = 26; 21.8%), a partially inclusive school setting (*n* = 19; 15.9%) and a specialized ASD school or center (*n* = 74; 62.18%). All the participants with ASD had received a formal diagnosis by either a pediatrician or a neurologist (using the DSM-IV-TR criteria and the Childhood Autism Rating Scale) prior to participating in the study. To verify the clinical diagnosis, as well as to determine the severity of the participants’ autistic symptoms, two parent report measures were administered, namely the Gilliam Autism Rating Scale, Third Edition (GARS-3) [44] and the Michigan Autism Spectrum Questionnaire (MASQ) [45]. Further, the Clinician-Rated Severity of Autism Spectrum and Social Communication Disorders (CRSASSC) measure [46] was used by the first author to independently assess the severity of the participants’ ASD symptoms.

The participants with ASD were intentionally recruited according to the following inclusion criteria. First, children who scored ≥ 71 on the GARS-3, which indicated that a diagnosis of ASD was very likely. Second, children with an IQ score of 70 or above, which ensured that any identified differences in performance were reflective of autistic symptomatology and not of general intellectual functioning. The IQ scores were obtained from the children’s school records. In the three countries of interest, a child’s IQ is normally determined by either a clinical psychologist or a school psychologist using the Arabic version of the Wechsler Intelligence Scale for Children, Third Edition (WISC-III; 1991). Third, children who scored 22 or above (i.e., the cut-off point for high functioning ASD) on the MASQ. Finally, children whose ASD was categorized as level 1 with regards to severity, which is characterized by the DSM-V as being “mild” and “requiring support.” The exclusion criterion for the ASD group was the presence of severe comorbidities (e.g., intractable epilepsy, severe self-injury, aggression, blindness and deafness). Children with blindness and/or deafness were excluded if their hearing and/or visual acuity had not been corrected to within normal limits.

The comparison (non-ASD or TD) group comprised 30 children with typical development (age range: 6–12 years; mean age = 9.06 years), who were all recruited from mainstream primary schools. Children with a history of any psychiatric, neurological or developmental disorders (as reported by their parents), a family history of ASD or a need to regularly use any psychotropic medication were excluded from the study. 

Due to the sizes of the two groups being unequal, a groupwise matching approach was used rather than a pairwise matching approach. This approach is in line with that used by Johnston, Murray, Spain, Walker and Russell [47]. It proved challenging to recruit children with typical development due to people’s general sensitivity with regards to data collection within Arab societies. Therefore, the ASD and TD groups were groupwise matched on the basis of the participants’ chronological age, gender (male or female), handedness (right or left, as assessed using a standard questionnaire; The Edinburgh Handedness Inventory [48]), non-verbal IQ (as measured using Raven’s Colored Progressive Matrices [RCPM] [49]) and parental education. There were no significant statistical differences (t-test/chi-square test) and no large effect sizes (Cohen’s d test) identified between the two groups in relation to any criteria relevant to the comparison variables, with all the *p* values being > 0.05. A summary of the group comparisons is presented in Table 1.

### 2.2. Instruments

#### 2.2.1. Screening Questionnaires

(1) Gilliam Autism Rating Scale – Third Edition (GARS-3 [44]).

The Arabic version of the GARS-3, has been translated and adapted for use in the Gulf region by the first author and was used after written permission was obtained from the Publisher (Pro-Ed). This normal referenced screening scale consists of 56 items that are based on the DSM-5 criteria for ASD. These items are grouped into six subscales: Restrictive; Social Interaction; Social Communication; Emotional Responses; Cognitive Style; and Maladaptive Speech. The GARS-3 is comprised of two indexes. For individuals who are non-verbal or suffer from a severe lack of communication skills, a four- subscale composite is used. A six-subscale composite is used for individuals who have verbal communication skills. The autism index is determined by summing the scores for each subscale. This scale is widely used to identify autism in individuals aged 3 to 22 as well as to estimate its severity. The parents of the children with ASD enrolled in the current study were able to complete the questions for this scale in 5 to 10 min for this study.

(2) Michigan Autism Spectrum Questionnaire (MASQ) [45].

This is a simple rating scale used to identify those with high functioning ASD. It consists of ten items. Each question has four potential responses, ranging from 0 to 4, yielding a total score of 30. The cut-off point is 22 or above for high functioning autism. The intermediate scores (14 through 21) predict autism. Although the scale focuses on the average age of the ASD group, ranging from 6 to 13 years, the authors did indicate that the scale may prove to be equally useful in older adolescents and young adults.

(3) The Clinician-Rated Severity of Autism Spectrum and Social Communication Disorders [46]: 

This scale is divided into the social and communication area and the restricted interests and repetitive behaviors area. Support levels are rated on a four-point Likert scale (level 0 = none, level 1 = requiring support, level 2 = requiring substantial support and level 3 = requiring very substantial support). This scale is used to assess the level of support that an individual need and then may guide the understanding of whether a person is ‘high functioning’ or more significantly impaired through the provided ratings: level 1 is mild, level 2 is moderate and level 3 is severe. The severity levels for each item are reported separately; a combined score for the overall severity should not be calculated.

#### 2.2.2. Assessment Measures

The following test instruments were used to assess the different domains of executive functioning. 

(1) The Behavior Rating Inventory of Executive Function, Second Edition (BRIEF-2; [42]).

The BRIEF-2 (Arabic Version, which is translated and adapted by the first author for use in the Gulf region, was used after obtaining written permission from Parinc (PAR)) is an updated version of a widely used measure of executive functioning that contains 63 items for assessing real-world executive functional behaviors in children and adolescents aged five to 18 years [42]. It is comprised of nine subscales (i.e., inhibit or inhibition, shift, emotional control, initiate, working memory, planning and organization, organization of materials, task monitoring and self-monitoring) with three index scores: the behavioral regulation index, emotional regulation index and cognitive regulation index. The BRIEF-2 also provides a Global Executive Composite (GEC). 

Using the BRIEF-2, parents are asked to rate their children’s behavior according to a three-point Likert scale (i.e., never, sometimes, often), with higher scores indicating greater EDF. Ratings are expressed as T-scores (*M* = 50; *SD* = 10) and higher scores are indicative of greater executive functioning difficulties. It should be noted that, for all the BRIEF-2 clinical scales and indices, T-scores ranging from 60 to 64 are considered to be mildly elevated, while T-scores ranging from 65 to 69 are considered to be potentially clinically elevated. Moreover, T-scores of or above 70 are considered to be clinically elevated. 

Psychometric Properties of the BRIEF-2 appear high with the internal consistency in the range of (αs = 0.76–0.97) and test-retest reliability for the indexes and composite were all above 0.80 over an average of 2.9-week time span [50]. The validity of the BRIEF-2 was determined based on the confirmatory factor analysis that supports the scale composition and the BRIEF three index structure. The BRIEF-2 is correlated with other measures of behavior and IQ, indicating the validity of the scale. The BRIEF-2 was able to discriminate among clinical groups [42]. 

(2) The Cambridge Neuropsychological Test Automated Battery (CANTAB; [43]).

In the present study, four subtests from the CANTAB computerized battery, namely the intra-dimensional/extra-dimensional set-shifting task, the spatial working memory task, the Stockings of Cambridge task and the stop signal test, were used to assess specific domains of executive functioning, that is, the mental flexibility function, the working memory, the spatial planning function and response inhibition (see Table 2 for definitions of the key outcome measures). The battery uses non-verbal tasks that are displayed on a screen and it requires non-verbal answers to be given on the same touchscreen. 

### 2.3. Procedures

This study was conducted in accordance with the ethical standards established by QUT’s University Human Research Ethics Committee. Written informed consent was obtained from all the participants’ parents/caregivers prior to the commencement of the study. Further, all the child participants verbally assented to participate. The evaluation procedure was divided into two sessions. First, during the parent interviews, the parents rated their children’s behavior using the BRIEF-2. Second, the CANTAB battery was individually administered to each child on two separate days. The sequence of the neuropsychological assessment was countered-balanced across the participants to exclude any order effect. Short breaks were allowed throughout each assessment session to ensure that the children remained motivated.

### 2.4. Analysis

All the statistical calculations described in this study were performed using SPSS software (version 23.0). In terms of the BRIEF-2, the cut-off criterion for the instrument (a score of 60 or above) was used to estimate the proportion of elevated T-scores (i.e., the proportion of participants who scored above the clinical cut-off point). Further, descriptive statistics (mean, standard deviation, minimum and maximum) were also calculated in relation to the standard scores for all the key variables investigated by the BRIEF-2. An independent samples t-test was performed to determine the extent of the variation in executive functioning performance seen between the children with ASD and the TD children on both the BRIEF-2 and the CANTAB battery. 

As a rule, running multiple comparisons in separate t-tests increases the likelihood of a type I error occurring (i.e., rejecting a null hypothesis [Ho] when Ho is true). Due to the large number of comparisons performed in the current study, the Bonferroni correction was used to control the family-wise error rate (FWER) and to avoid increasing the risk of a type I error. When calculating the Bonferroni adjustment, the alpha value (*p* = 0.05) is divided by the total number of planned comparisons being performed.

For the multiple comparison analysis of the nine clinical subscales of the BRIEF-2, the Bonferroni threshold was calculated by dividing the error probability (*p* = 0.05) by the number of subscales used to evaluate the various executive functioning domains (*n* = 9; 0.05/9 = *p* ≤ 0.006) and the four BRIEF-2 index scores (*n* = 4; 0.05/4 = *p* ≤ 0.013). 

Moreover, for the multiple comparison analysis of the four CANTAB subtests, the significance value was set at *p* = 0.0013.

To determine the meaningfulness of the group differences identified in the participants’ executive functioning abilities, the effect sizes (ES) were calculated using Cohen’s d [51].

Additionally, to investigate the effect of age on performance, a regression analysis was performed using age as the predictor variable and the raw scores for each child’s performance on each of the subscales as the outcomes of interest. Given that there were several predictor variables included in the regression analyses and that there was an increased risk of type I and II errors, we chose the more conservative p-value of 0.01 to indicate statistical significance.

## 3. Results

The results of this study are discussed in three stages according to the overall objectives of the study.

### 3.1. Behavioral Rating Scale (The BRIEF-2)

#### 3.1.1. The Profile Distribution and the Prevalence of the Executive Dysfunction Patterns among the Children with ASD

Table 3 presents descriptive statistics concerning the nine subscales, three indices and the Global Executive Composite (GEC) of the BRIEF-2 for the children with ASD.

As shown in Table 3, the highest means among the BRIEF-2 subscales for the ASD group were found for the Plan/Organize subscale (*M* = 63.47, *SD* = 6.94), followed by the Working Memory, Emotional Control, Self-Monitor, Initiate and Shift subscales. Further, the lowest means were found for the Organization of Materials subscale (*M* = 54.36, *SD* = 5.32), followed by the Task-Monitor and Inhibit subscales. Although the mean scores for the Inhibit, Task-Monitor and Organization of Materials subscales were elevated above the mean score of 50, they were not in the mildly elevated range of 60–64.

Similar results were found for the three indices, with the most significant deficits being identified in relation to the Emotion Regulation index (ERI: *M* = 62.59, *SD* = 7.36), followed by the Behavioral Regulation and Cognitive Regulation indices (BRI: *M* = 61.32, *SD* = 7.15 and CRI: *M* = 61.21, *SD* = 6.27) for the children with ASD, which reflects global executive dysfunction. The overall index, namely the GEC, was also found to be clinically elevated in the ASD group (GEC: *M* = 64.21, *SD* = 6.89), which suggests the presence of executive dysfunction in the everyday behavior of the children with ASD, as reported by their parents. 

Table 4 below outlines the proportions of the t-score elevations for the different BRIEF-2 domains.

Table 4 shows the percentages of the children with ASD who exhibited t-score elevations of 60, 65, 70 or above for each scale and index of the BRIEF-2. In terms of the subscale scores, nearly 75% of the sample surpassed the clinical cut-off point for the BRIEF-2 Plan/Organize subscale. Moreover, the children with ASD (62.2%) exhibited the second highest elevation in relation to the Emotional Control scale. An examination of the base rates displayed in Table 4 reveals that 60.5% of the sample were rated by their parents as having T-scores of 60 or above for the Self-Monitor subscale and 59.7 for both the Shift and the Working Memory subscales. In contrast, close to half (45%) of the sample surpassed the clinical cut-off point for the Initiate scale.

A similar pattern of results was found in relation to the index scores. Some 68.1% of the children with ASD had Emotional Regulation t-scores of 60 or above, whereas 54.6% and 52.1% of the children with ASD scored above the clinical cut-off points for the Cognitive Regulation and Behavioral Regulation indices, respectively. The results shown in Table 4 also indicate that a greater proportion of children with ASD (75%) demonstrated significant elevations in the GEC.

#### 3.1.2. Group Differences Based on the BRIEF-2 Raw Scores

Table 5 presents the group-related differences in terms of the executive functioning performance of the children with ASD and the TD children. The results of the independent samples t-test suggested that the children with ASD displayed more executive functioning difficulties across all the relevant subscales of the BRIEF-2 than the TD children. All the identified differences are significant at the *p* < 0.01 level, with the large effect sizes concerning the nine subscales ranging from Cohen’s d = 1.76 to 2.53. The Plan/Organize domain had the largest effect size. Similarly, significant differences were found in relation to the three indices and the GEC, yielding Cohen’s d values in the 1.91 to 2.86 range.

### 3.2. Individual CANTAB Test Scores

Table 6 reports the mean performances (raw scores) of the participants on the CANTAB tests for each delineated executive functioning domain, namely the I/ED, SWM, SOC and SST domains, as measured by group. The results concerning each CANTAB subtest are discussed in the following:

#### 3.2.1. Flexibility/Switching Task 

With regards to the mental flexibility/shifting component, the variable, namely the total errors adjusted, was examined during the task. There was a significant difference found between the ASD and control groups in relation to the ID/ED variable, that is, in relation to the total errors adjusted (*p* < 0.05; Cohen’s d = 1.17). The effect size of this difference was large.

#### 3.2.2. Working Memory Task 

As for the working memory domain, the between errors variable was examined. A significant difference was found between the ASD and control groups with regards to the SWM between errors (*p* < 0.05; Cohen’s *d* = 1.62). The effect size (d) indicated large significant differences for this test.

#### 3.2.3. Planning Task 

In terms of the planning function, no significant difference was found between the two groups based on the t-test for planning (*p* > 0.05), which included the SOC minimum moves criterion.

#### 3.2.4. Response Inhibition Task 

In the test of inhibition, the stop signal reaction time (SSRT) was the key variable analyzed. There were significant differences found between the ASD and control groups in relation to inhibition (*p* < 0.05; Cohen’s d = 1.13), reflecting a large effect size.

### 3.3. Relationship between the Executive Functions and Age

#### 3.3.1. Age Differences in Relation to the BRIEF-2.

We used a linear regression analysis to examine whether age significantly predicts the scores for the BRIEF-2 subscales. The age variable was entered as a continuous variable. The regression model results concerning the BRIEF-2 subscales are summarized in Table 7. In line with the approach of prior studies investigating age-related differences in executive functioning on the part of those with ASD [31,38], this study focused on raw scores rather than on T-scores.

The linear regression results indicated that the chronological age variable did not predict the discrepancy scores in relation to the BRIEF-2 subscales, save for in the case of the Plan/Organize subscale (F = 9.633, *p* < 0.01), for which age accounted for only around 7% of the variance. The beta (β) or standardized regression coefficient for age was significant at the *p* < 0.01 level. Although age was found to be a statistically significant contributor, the effect was only relatively minor. More specifically, 93% of the variance was accounted for by other factors, including errors. As might be expected, the children’s planning skill become less impaired as they grew older.

Overall, the results of this study provide evidence that age does not account for a significantly large percentage of the variance in the executive functioning of children with ASD, as measured in relation to real-life situations.

#### 3.3.2. Age Differences in Relation to the Individual CANTAB Tasks.

The results regarding the age-related differences identified in relation to the four utilized CANTAB tasks are presented in Table 8. Separate regression models were used for the individual measures of executive functioning.

As can be seen from Table 8, the regression analyses revealed that age did have an effect on the total errors adjusted in relation to the CANTAB ID/ED set-shifting task (β = 0.36, *p* < 0.001), accounting for some 12% of the variance in this component of executive functioning. This finding suggests that the children with ASD tended to make less errors on the flexibility task as they grew older. 

A significant age-related effect was also found in terms of the between-errors outcomes for the CANTAB spatial working memory task (β = −0.37, *p* < 0.001), accounting for 13% of the variance in performance. This indicates that as the age of the participants with ASD increased, the number of between-search errors during the spatial working memory task decreased.

In terms of the planning function during the SOC task, chronological age emerged as a significant predictor of the discrepancy in scores in relation to this task. The β or standardized regression coefficient for age was significant (β = 0.32) at the *p* < 0.001 level, indicating that as the participants’ age increased, the average number of moves needed to solve the tasks decreased. Although age was found to be a statistically significant contributor, the effect was relatively small (Ad*R*^2^ = 0.09). 

With regards to inhibition, similar results were found concerning the effect of age in relation to the CANTAB SSRT task, indicating that the ability to inhibit prepotent responses may improve slightly with age. However, while the standardized regression coefficient was statistically significant, age made only a minor contribution to it.

Overall, the findings of the regression analysis indicated that the contribution of chronological age to the variance in the participants’ performance during the utilized executive functioning tasks, as measured in a laboratory setting, was significant and thus should not be ignored.

## 4. Discussion

In general, the findings of the study revealed significant deficits on the part of children with ASD in relation to some, albeit not all, aspects of executive functioning, as revealed by both the behavioral ratings and the performance-based measures. This suggested that the identified executive functioning deficits are specific rather than global. The key findings of this study will now be discussed in relation to the overall objectives of the research.

### 4.1. Global Profiles of Executive Functioning

The findings of this study suggest that, when compared to the normative standards used to evaluate the EFs and/or to the performance of TD children from the Gulf region, the children with ASD exhibited elevated executive functioning problems, as reported by their parents using the BRIEF-2. The findings are hence consistent with both our expectations and the results of prior studies that used the original BRIEF to report significant EF deficits in individuals with ASD [37,52,53,54]. The deficits consistently exhibited by individuals with ASD during everyday life could be attributed to the fact that it is relatively rare for daily tasks to have a clear structure and explicit instructions, which contributes to the general complexities of everyday life [55]. Further, there appears to be a trend for children with ASD to demonstrate impairments when performing tasks that require the generation of multiple responses [56]. Although group differences have been found to exist between children with ASD and TD children (i.e., those within the normal expected range), not all individuals with ASD encounter problems in one or more executive functioning domains. A likely explanation for these findings concerns the heterogeneity of the EF problems seen in individuals with ASD, which indicates the importance of focusing on individual differences when studying EF in those with ASD [38].

The majority of the prior research involving the BRIEF and the BRIEF-2 has indicated that the most profound executive functioning deficit found across the ASD population is seen in relation to the shift (i.e., flexibility) subscale, which measures cognitive flexibility and transitioning, when compared to the other subscales [37,42,53,54,57,58]. However, the findings of the present study suggest this may not always be the case. While this study also found that performance (as measured on multiple subscales) was impaired, the planning/organization subscale was found to reveal the most salient dysfunction in children with ASD.

This finding is arguably somewhat uncharacteristic of individuals with ASD, as they commonly display the most profound executive functioning deficit in relation to the flexibility/shifting subscale. This difference between the findings of the present study and those of prior investigations could reflect cultural differences in terms of the caregivers’ priorities and expectations regarding their children. Planning problems can easily be perceived by caregivers, particularly in non-test scenarios, since they typically result in the noticeable avoidance of taking action and in difficulty following daily routines and completing school projects. Indeed, the parents of children with ASD often complain that if they did not assist with daily routines or projects for school, then such things would not get done [59]. Furthermore, negative parenting approaches could have an indirect adverse effect on children’s executive functioning. In fact, parenting behavior has been suggested to be a key environmental determinant of children’s executive functioning [60]. Authoritarian parenting represents a common approach to parenting in the Gulf region. Based on the first author’s experience, parents there tend to emphasize a sort of blind obedience and seek compliance from their children, which actually places the children at risk of developing difficulties with planning and becoming overly reliant on their caregivers when it comes to taking action and making decisions. Hence, this parenting approach may hinder children’s ability to make their own decisions, to consider their actions based on the available alternatives and to explore the consequences of future behavior. It can actually severely damage their sense of autonomy as well as their ability to solve problems and to plan ahead. 

Further, the findings of this study revealed the second most significant area of weakness was found to be the emotional control subscale, followed by the self-monitor, shift, working memory and initiation subscales. Although performance on the organization of materials, task-monitor and inhibit subscales was found to be elevated relative to the normal range in the ASD group, it did not surpass the clinical cut-off point for any of them. Clearly, individuals with ASD do experience EF problems, albeit not always in the same areas [25]. Nonetheless, this study extended the prior literature by indicating the rank order of performance for the various EF domains.

Despite the BRIEF-2 having been shown to capture real-world behavioral manifestations of executive dysfunction through informant reporting, the results regarding EDF when the scale is used with an ASD population should be interpreted with caution. One possible explanation for the high scores of children with ASD when compared to the TD children is parental bias, since the parents of individuals with ASD may either over or under report their child’s current symptoms [61]. However, it must also be recognized that the BRIEF-2 does not provide single-label domains and instead intermixes the rating items for each domain, which helps to minimize the reporting bias [62].

### 4.2. The CANTAB

The results of this study indicated significant differences in the EF performance on the CANTAB battery between the children with ASD and those with typical development across each EF component, with the exception of the planning component. This section focuses on the findings related to each of the four domains of EF in children with ASD. 

#### 4.2.1. Flexibility

This study examined the set-shifting abilities of children with ASD by assessing their total number of errors on the ID/ED shift test. The participants with ASD exhibited set-shifting deficits. The findings were hence similar to those of certain previous studies that used either the same ID/ED shift task measurement [63,64,65] or other flexibility tasks, such as the WCST [66,67,68,69,70]. Such deficits in flexibility were also found in children with ASD when compared to children with other neurodevelopmental disorders, including ADHD [14,16]. One possible psychological explanation for poor performance on the ID/ED task is difficulty shifting attention away from the previously relevant stimulus and toward the newly relevant stimulus due to the inhibition of the previously reinforced dimension [71,72]. Such deficits in reversal shift may reflect difficulties in adjusting one’s thoughts or actions in response to situational changes. 

However, several flexibility studies that employed the ID/ED task with an ASD population found no differences between the ASD group and the typically developing population [29,31,73,74,75]. Some researchers (e.g., Cantio and colleagues (2016)) have suggested that the unimpaired performance of individuals with ASD in terms of the flexibility function may be due to the fact that such individuals perform better on computerized versions of tasks than on human-administered tasks [29]. However, this claim cannot currently be fully verified because prior studies using this task have yielded inconsistent findings. One explanation for the findings relative to the lack of a group difference in the ID/ED task is attributable to the fact that the ID/ED task may not be sufficiently sensitive to detect cognitive flexibility deficits.

#### 4.2.2. Working Memory 

With regard to the working memory (WM) task, our findings revealed SWM difficulties in the ASD group. This is in line with the findings of certain prior studies that also reported impairments in the SWM abilities of those with ASD using either the CANTAB SWM task [14,65,76] or other comparable tasks [77,78]. In contrast, Ozonoff and Strayer (2001), who used three computerized WM tasks, did not identify a significant deficit in the WM performance of individuals with ASD [79]. The deficit in the WM domain may hence be explained by the type of WM task used. Such tasks increase the amount of information that needs to be remembered and the number of trials over which it needs to be retained [76]. However, some studies involving the CANTAB indicated the absence of spatial WM deficits in ASD groups [29,31,73]. We recognize that homogeneity exists within the ASD population in terms of the phenotype and neurocognitive features, which can influence test results.

#### 4.2.3. Planning

As for planning, this study did not find a significant difference between the groups (ASD and control) regarding the number of problems solved using the minimum number of moves. This absence of a group difference concerning the main outcome measure of the planning test was quite surprising, since the planning domain has consistently been found to be impaired in individuals with ASD [9,38,67]. The present findings regarding unimpaired planning in children with ASD are consistent with those of prior studies that used the same instrument [14,29,74]. However, these findings contradict those of studies that used traditional planning tasks and reported impaired planning abilities in children with ASD [27]. This discrepancy could be attributed to the performance mode. There is a trend for individuals with ASD to perform worse on standard human-administered neuropsychological tasks (e.g., the Tower of London task) than on their computer-administered variants (e.g., the Stockings of Cambridge subtest) [80]. Yet, this cannot be wholly accurate, since the present study showed that individuals with ASD did not exhibit superior performance on all computer-based tasks. Further, Williams and Jarrold (2013) failed to identify a difference in performance between the human and computerized administration of the Tower of London task among individuals with ASD [81]. Other studies that employed the same instrument described reduced planning abilities in those with ASD [16,63,67,75], which indicates that the absence of a difference in planning performance between the ASD and control groups in this study might not be due to the performance mode.

According to Robinson et al. (2009), an alternative explanation for the disparity between the results of studies that used traditional tests and those that applied computerized tests may concern how the participants were instructed to complete the task [27]. When using computerized versions, additional information is presented to participants, including the minimum number of moves required to construct the desired pattern [27]. The provision of such information may enhance the planning performance of children with ASD by reducing the prepotent response or restricting the number of moves needed to construct the pattern [27]. This contrasts with traditional tests, wherein participants are only instructed to construct the desired pattern in as few moves as possible [27]. Nonetheless, the present results extend those of previous empirical studies regarding the relatively unimpaired planning skills of those with ASD when compared to the other EF domains. Further, the results are consistent with the literature indicating that the performance of individuals with ASD may be impaired in relation to certain EF components but not in relation others. This is also the case for some tasks but not for others. 

#### 4.2.4. Inhibition

During the inhibition task, the ASD group appeared more impaired than the control group regarding the SSRT variable of the SST. To the best of our knowledge, no prior studies have employed the CANTAB SST to measure the inhibition responses of children with ASD, although a deficit in response inhibition is one EF problem commonly observed in such children [14,27,69]. Yet, other studies failed to detect an inhibition deficit, especially those that applied the Stroop task [68,82,83,84]. The present findings are consistent with previous evidence of response inhibition derived from studies that used alternative tests, such as the SSRT of the change task, which measures prepotent response inhibition [85]. Impairments in inhibition may stem from the arbitrary rules that require individuals to inhibit a highly automatic response and, therefore, cause particular difficulties for individuals with ASD [55,86,87].

Some prior studies have reported unimpaired inhibition on the SST among children with ASD when compared to typically developing children [88]. Thus, there remains some ambiguity concerning whether or not children with ASD show impairments in inhibition [86,87]. Some studies have indicated that individuals with ASD only exhibit inhibition problems when a prepotent response is involved [11,54,82], whereas more recent studies have confirmed that children with ASD exhibit impaired resistance to distractor inhibition while maintaining unimpaired prepotent response inhibition [88]. These contradictory results may be influenced by the type of task used and the type of inhibition involved. This underlines the importance of using multiple measures to assess a putative single cognitive ability [89]. 

These findings extend the literature by providing evidence of the impairments seen in children with ASD regarding the response inhibition function, as assessed using the CANTAB SST, which is here employed for the first time to assess an ASD population.

### 4.3. The Relationship Between Age and Executive Functioning

The third aim of this study was to investigate whether executive functioning abilities are distinct across age in participants with ASD. Based on the BRIEF-2, the results provided limited evidence of age-related differences among the children with ASD. No significant age-related differences were found with regard to almost all BRIEF-2 subscales. Age-related differences were only found in relation to the planning subscale. This suggests that the participants’ executive dysfunctions, as experienced in everyday life settings, remained relatively stable as they aged. Such a finding regarding the lack of a relationship between age and everyday executive functioning raises an important question concerning the ecological appropriateness of the BRIEF-2 to measure executive functioning in daily life from a developmental perspective.

These results regarding the lack of the chronological effect on EF are in line with those of studies suggesting the stability of EDF in children with ASD [35,36,37]. However, the present results contrast with those of previous reports on typical development, which indicated age-related declines in problematic EF behaviors during childhood and adolescence [90]. In fact, few studies have investigated potential developmental differences in EF in those with ASD using the chosen rating scale (i.e., the BRIEF). Van den Bergh et al. (2014) found limited evidence of age-related differences in children with ASD in relation to inhibition and planning. Fewer inhibition problems and more planning problems were found for older children with ASD. Age-related differences were not found in terms of the WM or cognitive flexibility [38]. Rosenthal et al. (2013) found widening age-related differences in EF. Their results showed that, when using a parent report (i.e., the BRIEF), EF appeared to become increasingly problematic over time. Flexibility remained particularly impaired across the age groups in those with ASD [37]. The failure to identify age-related gains in almost all the BRIEF-2 components may be attributed to the fact that the nature of EDF in children with ASD remains relatively stable throughout childhood [27].

During the CANTAB tasks, we noted age-related improvements in EF aspects, namely cognitive flexibility, WM, planning and inhibition. In the ID/ED task, children with ASD exhibited a significant relationship between shifting performance and age, resulting in a reduction of errors. In the SWM task, children with ASD also exhibited a significant relationship between age and the number of errors made during the SWM, indicating that maintaining spatial information increased with age. In the SOC, there were significant correlations between the performance of the planning task used and age indicated an increase in the number of problems the child solves with the minimum number of moves, reflecting development in strategic planning skills in the ages assessed. In the SST task, children with ASD exhibited significant relationships between inhibition, performance and age; this indicated an increase in the speed of inhibition responses. 

The present results regarding age-related improvements in EF are consistent with those of some prior studies, which suggested age-related gains in EF [31,32]. However, in terms of chronological changes in EF, the present results do not support the suggestion that executive deficits worsen with age [38]. The results also partly contrast with the lack of age-related improvements in EF reported by Ozonoff et al. (2004) [64]. 

According to Happé et al. (2006), a possible explanation for the identified age-related differences could be educational interventions or individual compensation strategies [31]. However, this interpretation regarding educational interventions is not applicable to the context in which the present study was conducted, since the emphasis of both assessment and treatment was on the behavioral features rather than the neurological basis of ASD. Research regarding EDF in those with ASD is still in its infancy. Additionally, Arabic versions of the standard instruments used to evaluate EF are not available. An alternative explanation for the change in the nature of the deficit in some individuals’ EF is that the development trajectory of the executive functions (for people with ASD) runs parallel below the trajectory of TD individuals [80]. Further, the CANTAB may be sufficiently sensitive to detect the EF maturation of the relevant brain structures. Given the cross-sectional nature of our study, longitudinal studies are also required to advance our knowledge regarding chronological changes in EF.

## 5. Conclusions

This study found that children with ASD experience significant EF difficulties in both everyday and laboratory settings, albeit not in the same areas. For the parent rating of EF, 75% of children with ASD surpassed the clinical cut-off points for the Global Executive Composite. The most prominent deficits were found in relation to the BRIEF-2 planning subscale. The findings of the laboratory-based EF assessment tools suggest significant impairments in the flexibility, working memory and inhibition of the children with ASD, although they exhibited preserved performance for the planning function. The divergence between the informant measures and the performance-based findings could reflect a poor overlap between the two approaches. This study showed that the divergences in the age-related patterns of performance on the CANTAB widened, while they narrowed on the rating scale.

The present study represents an important extension of the literature, since it produced new neurobehavioral information regarding the EF of school-age children with ASD in the Gulf region. Additionally, it performed an extensive evaluation of EF, including a performance-based measure (CANTAB) and an ecologically valid measure of everyday EF (BRIEF-2), to obtain information regarding the higher-order cognitive functions of this population. These are key strengths of this study. Another strength concerns the sample size, which was relatively large for a study involving individuals with ASD. 

However, it is important to acknowledge that the present study did have a number of limitations. First, the rate of refusal to participate in the study on the part of children with typical development and their families was high. The parents appeared worried that the resulting feedback concerning their children might confirm the presence of executive dysfunctions. It must be recognized that certain dysfunctions can prove stigmatizing for a child and his/her family in Arab culture; therefore, many parents did not want their children to participate in the study. Thus, the population with typical development included in the present study cannot be deemed representative of the entire population. Second, due to the lack of standardized data concerning the BRIEF-2 in the Gulf region, it proved difficult to recruit a suitable number of participants for a matched control group. This was compensated for by using a cut-off T-score of 60, which indicated executive dysfunction according to the original norms. Third, only children with mild/high functioning ASD were included in the present study; therefore, the findings may not generalize to a wider ASD population. 

A further limitation that must be borne in mind relates to the fact that, with regard to the matching of participants, our analysis revealed no significant differences between the TD and ASD participants in terms of their non-verbal IQ scores (*p* = 0.06). Yet, it must be recognized that matching based on a lack of significance at α = 0.05 can prove problematic due to the increased likelihood of a type II error occurring, as denoted by the beta error (accepting Ho when it should be rejected) being unacceptably high for the marginal approach (e.g., *p* = 0.06), which is borderline nonsignificant at *p* = 0.05. For this reason, it is important to acknowledge that the findings of this study could be attributed to differences in the participants’ IQ scores.

Lastly, a cross-sectional approach was used to examine the age-related differences in EF among the participants. Therefore, the results regarding the effects of chronological age must be interpreted with caution. Future studies could apply a longitudinal approach to better determine the developmental trajectories of EF in individuals with ASD.

Our future research will examine whether the executive functions are inter- or intra-dependent on each other. Examining both the collinearity and the explanatory power of each subtest will allow for a better understanding of the ways in which the distinct executive functions relate to one another. It will also assist with investigating whether unique neuropsychological profiles can be observed for clinical and non-clinical groups. Such an examination should prove particularly helpful for practitioners and researchers seeking to move beyond the deficit narrative when it comes to those with ASD and toward a narrative that gauges the unique cognitive profiles of individuals with ASD, as well as the unique strengths exhibited by TD children, while still focusing on areas of need.

Despite the above-mentioned limitations, the findings of this study have important research and clinical implications concerning the profiling and targeting of the executive functions during the assessment and treatment process associated with ASD. These implications include identifying individual differences in EF among children with ASD as well as attempting to understand their individual strengths and weaknesses. Further, measures of EF could be used to guide the diagnosis of ASD, while knowledge regarding the specific profiles of the cognitive dysfunctions associated with ASD could assist with the design of cognitive interventions and the development of more appropriately targeted treatment approaches.

## Figures and Tables

**Table 1:** Participants’ Demographic Characteristics and Descriptive data for the group comparisons.

Comparison Criteria	Target Group (*n* = 119)		Control Group (*n* = 30)		Significance Tests			
	Children with ASD		TD Children					
**Continuous Data**	***M***	***SD***	***M***	***SD or %***	**t**	***p***	**d**	**Adequacy**
Age	8.72	1.96	9.06	1.42	1.05	0.71	0.21	Matched
Non-verbal IQ ^*^	29.76	1.92	29.80	2.64	0.69	0.060	0.17	Matched
**The categorical variables**	***N***	**%**	***N***	**%**	**χ2**	***p***	**d**	**Adequacy**
Gender	M = 95	79.8	M = 24	80.0/20.0	0.00	0.98	-	Matched
	F = 24	20.2	F = 6					
Handedness	R = 104	87.4	R = 23	76.7/23.3	2.191	0.14	-	Matched
	L = 15	12.6	L = 7					
Father’s Education Level (% Secondary/College degree)	67/52	56.3/43.7	16/14	53.3/46.7	0.09	0.77	-	Matched
Mother’s Education Level	78/41	65.5/34.5	20/10	66.7/33.3	0.01	0.91	-	Matched

Key: *M* = Mean; *SD* = Standard Deviation; *n* = % of total. * Continuous data were analyzed using the t-test, while the categorical variables were analyzed using the χ^2^ test. d values are ESs of ASD versus typically developing children (TDC). The possible raw scores for the test ranged from 0 to 36.

**Table 2:** Definitions of the key outcome measures of the Cambridge Neuropsychological Test Automated Battery (CANTAB).

Constructs	Task Name	Outcome Variable	Definition
Shift	Intra-Extra-Dimensional Shift (IED)	Total errors adjusted	The total number of times that the subject chose a stimulus incompatible with the current rule, plus, for each problem that was not reached (if any), an adjustment is made to the score.
Working memory	Spatial Working Memory (SWM)	Between errors	The total number of times the subject revisits a box in which a token has previously been found in the same problem (calculated for assessed problems only).
Plan	Stockings of Cambridge (SOC)	Problems solved in minimum moves	The number of times the subject has successfully completed a problem in the minimum possible number of moves.
Inhibit	Stop Signal Task (SST)	Stop signal reaction time (SSRT)	The length of time between the go stimulus and the stop stimulus at which the subject is able to successfully inhibit their response on 50% of trials.

**Table 3:** Descriptive Statistics Concerning the Executive Functioning Domains Across the Scales, Indices and Global Executive Composite (GEC) of the BRIEF-2.

BRIEF-2	ASD Sample (*n* = 119)					95% Confidence Interval for Mean	
Subscales	Min	Max	*M*	*SD*	SE	Lower	Upper
Inhibit	47.0	85.0	59.83	8.54	0.78	58.28	61.38
Self-Monitor	49.0	78.0	62.07	6.70	0.61	60.85	63.28
Shift	46.0	79.0	61.24	6.64	0.61	60.03	62.44
Emotional Control	47.0	84.0	62.12	9.10	0.84	60.47	63.77
Initiate	47.0	79.0	61.36	7.19	0.66	60.06	62.67
Working Memory	43.0	83.0	62.65	9.22	0.85	60.97	64.32
Plan/Organize	50.0	77.0	63.47	6.94	0.64	62.21	64.73
Task-Monitor	46.0	73.0	57.06	7.35	0.68	55.73	58.39
Organization of Materials	47.0	70.0	54.36	5.33	0.49	53.39	55.32
**Indices**							
Behavior Regulation Index (BRI)	48.0	84.0	61.33	7.15	0.66	60.03	62.63
Emotion Regulation Index (ERI)	47.0	79.0	62.60	7.37	0.68	61.26	63.93
Cognitive Regulation Index (CRI)	50.0	77.0	61.21	6.27	0.58	60.07	62.35
**Global Executive Composite (GEC)**	51.0	84.0	64.22	6.89	0.63	62.97	65.47

All the scores are reported as T-scores (*M* = 50, *SD* = 10), with T-scores between 60 and 64 being considered to indicate mild elevation, T-scores between 65 and 69 considered to indicate potential clinical elevation and T-scores of 70 or above considered to indicate clinical elevation. Likert scale range from 1–3, with higher scores indicating poorer/worse executive functioning. (SE): The standard error.

**Table 4:** The Parent-Rated BRIEF-2 Base Rates for the Elevated T-Scores for the Children with autism spectrum disorder (ASD).

BRIEF-2	T-Score Elevation			
Subscale	60–64 * Mild Elevation	65–69 * Potential Clinical Elevation	≥70 Clinical Elevation	T-Score Elevation
Inhibit	13.4	18.5	14.3	46.2
Self-Monitor	22.7	34.5	3.4	60.0
Shift	29.4	20.2	10.1	59.7
Emotional Control	21.8	12.6	27.7	62.2
Initiate	17.6	11.8	16.0	45.4
Working Memory	19.3	16.0	24.4	59.7
Plan/Organize	30.3	26.1	18.5	74.8
Task-Monitor	13.4	19.3	3.4	36.1
Organization of Materials	15.1	0.8	3.4	19.3
**Indices**				
(BRI)	21.0	15.1	16.0	52.1
(ERI)	26.1	24.4	17.6	68.1
(CRI)	24.4	17.6	12.6	54.6
(GEC)	32.8	18.5	23.5	74.8

* The percentage impaired (%) indicates the proportion of participants with ASD with a score considered to have potential clinical significance (a T-score ≥ 60 for the BRIEF-2).

**Table 5:** Comparing the ASD and typically developing (TD) Groups Based on the BRIEF-2 Scales.

BRIEF-2	ASD Sample (*n* = 119)		Control Group (*n* = 30)		T-Statistic	Bonferroni Adjusted *p*-Value	D
	*M*	*SD*	*M*	*SD*			
**Subscales**							
Inhibit	15.75	2.91	11.27	1.72	10.875 **	0.006	1.87
Self-Monitor	8.71	1.37	5.83	1.26	10.397 **	0.006	2.18
Shift	14.66	2.06	10.80	1.83	9.382 **	0.006	1.98
Emotional Control	16.04	3.39	11.77	2.81	6.375 **	0.006	1.37
Initiate	10.48	1.72	6.70	1.56	10.943 **	0.006	2.30
Working Memory	17.02	3.40	10.37	2.06	13.626 **	0.006	2.36
Plan/Organize	18.08	2.66	11.57	2.47	12.167 **	0.006	2.53
Task-Monitor	10.61	1.96	7.3	1.78	8.427 **	0.006	1.76
Organization of Materials	11.03	1.65	8.07	1.59	8.874 **	0.006	1.82
**Indices**							
BRI	24.45	3.64	17.10	2.33	13.628 **	0.013	2.40
ERI	31.02	4.89	22.57	3.88	8.795 **	0.013	1.91
CRI	67.23	8.71	44.00	7.98	13.265 **	0.013	2.78
GEC	122.70	14.63	83.67	12.49	13.421 **	0.013	2.86

** *p* < 0.01 according to the independent samples t-test. Likert scale range from 1–3, with higher scores indicating poorer/worse executive functioning.

**Table 6:** Comparing the ASD and control groups based on the CANTAB test scores.

Individual CANTAB Test Scores	ASD Group (*n* = 119)		TDC Group (*n* = 30)		*t*-Statistic	Bonferroni Adjusted *p*-Value	*D*
	*M*	*SD*	*M*	*SD*			
**Flexibility/Shifting**							
*Intra-Extra-Dimensional Shift (IED)*							
Total errors adjusted	22.05	13.33	10.77	2.97	8.441 *	0.013	1.17
**Working Memory**							
*Spatial Working Memory (SWM)*							
Between errors	59.50	11.90	39.17	13.11	8.19 *	0.013	1.62
**Planning**							
*Stockings of Cambridge (SOC)*							
Problems solved in minimum moves	7.97	1.94	8.57	1.38	−1.591	ns	0.36
**Response inhibition**							
*Stop Signal Task (SST)*							
Stop signal reaction time ^†^	608.80	192.53	440.00	86.13	7.141 *	0.013	1.13

* *p* < 0.05. ^†^ The SSRT score is given in milliseconds; ns: no significance.

**Table 7:** Linear Regression Predicting the BRIEF-2 Scores by Age for the Children with ASD.

Subscale	β	t	*p*	*R* ^2^	Ad*R*^2^	F
Inhibit	0.026	−0.279	0.781	0.001	0.008	0.078
Self-Monitor	0.210	−2.327	0.022 *	0.044	0.036	5.413
Shift	0.013	−0.136	0.892	0.000	−0.008	0.019
Emotional Control	0.115	−1.247	0.215	0.013	0.005	1.555
Initiate	0.161	−1.770	0.079	0.026	0.018	3.133
Working Memory	0.105	1.144	0.255	0.011	0.003	1.309
Plan/Organize	−0.276	−3.104	0.002 **	0.076	0.068	9.633
Task-Monitor	−0.041	−0.449	0.654	0.002	−0.007	0.202
Organization of Materials	−0.005	−0.054	0.957	0.000	−0.009	0.003
**Behavioral Section**						
BRI	−0.100	−1.088	0.279	0.010	0.002	1.184
ERI	−0.086	−0.928	0.355	0.007	−0.001	0.862
CRI	−0.167	−1.836	0.069	0.028	0.020	3.370
GEC	−0.153	−1.675	0.097	0.023	0.015	2.805

* *p* < 0.05, ** *p* < 0.01.

**Table 8:** Linear Regression Predicting the CANTAB Scores by Age for the Children with ASD.

CANTAB Test Scores	β	t	*p*	*R* ^2^	Ad*R*^2^	F
**Flexibility** *(ID/ED)*						
Total errors adjusted	−0.355	−4.114	0.000 ***	0.126	0.119	16.924
**Working Memory** *(SWM)*						
Between errors	−0.37	−4.317	0.000 ***	0.137	130	18.633
**Planning** *(SOC)*						
Problems solved in minimum number of moves	0.318	3.632	0.000 ***	0.101	0.094	13.195
**Inhibition** *(SST)*						
Stop signal reaction time	−0.291	−3.291	0.001 **	0.085	0.077	10.830

** *p* < 0.01, *** *p* < 0.001.
